# Editorial: Superhalogens & superalkalis: Exploration of structure, properties and applications

**DOI:** 10.3389/fchem.2022.1075487

**Published:** 2022-11-15

**Authors:** Ambrish Kumar Srivastava, Iwona Anusiewicz, Suzana Velickovic, Wei-Ming Sun, Gennady L. Gutsev

**Affiliations:** ^1^ Department of Physics, Deen Dayal Upadhyaya Gorakhpur University, Gorakhpur, India; ^2^ Department of Chemistry, University of Gdansk, Gdansk, Poland; ^3^ Department of Physical Chemistry, Vinĉa Institute of Nuclear Science, University of Belgrade, Belgrade, Serbia; ^4^ Department of Basic Chemistry, School of Pharmacy, Fujian Medical University, Fuzhou, China; ^5^ Department of Physics, Florida Agricultural and Mechanical University, Tallahassee, FL, United States

**Keywords:** atomic clusters, superalkali, theory, superatoms, superhalogen

Atomic clusters (Srivastava et al., 2021), containing a few to a hundred of atoms, are intermediates between a molecule and bulk materials. Superatoms are a special class of atomic clusters, mimicking the properties of atom. Superhalogens are superalkalis are two examples of superatoms, which possess higher electron affinity than halogen and lower ionization energy than alkali atoms, respectively. The Research Topic “*Superhalogens & Superalkalis: Exploration of Structure, Properties and Applications*” provides a compendium of the recent research and development in this field. This Research Topic concludes two review articles and seven research articles.

Srivastava and Srivastava reviewed the application of superalkalis in the activation of carbon dioxide. Owing to low ionization energy, superalkalis become reducers and can easily transfer an electron to CO_2_, which possesses no positive electron affinity. They have discussed the CO_2_ reduction by various types of superalkalis such as typical superalkalis, binuclear superalkalis, special superalkalis, non-metallic superalkalis. These superalkalis differ in design based on different electron-counting rules. The authors also included the successive activation of six CO_2_ molecules using hexalithiobenzene (C_6_Li_6_), a closed-shell molecule with low ionization energy. Superalkalis have been substantially explored over the past couple of decades. In the review of Pandey et al., the recent developments in the theoretical design and characterization of a variety of superalkali-based compounds and their potential applications have been enumerated. They unveiled the potential applications of some novel superalkalis for capturing and storing CO_2_/N_2_ and also analyzed the first-order hyperpolarizability-based nonlinear optical (NLO) responses features of fullerene-like superalkali-doped B_12_N_12_ and B_12_P_12_ nanoclusters with good ultraviolet (UV) transparency and superalkali-based CaN_3_Ca system, a high sensitivity alkali-earth-based aromatic multi-state NLO molecular switch, and so on. They have also highlighted the interactions of superalkalis in gas and liquid phases in this review. The authors expected that their review will provide new insights on the possibility of expanding both the experimental synthesis and the practical use of superalkalis and related species.

Ye et al. designed the X@3^6^adz complexes by embedding X (=H, B, C, N, O, F, and Si) into the 3^6^adamanzane (3^6^adz) complexant. It is interesting to find the low adiabatic ionization energies (AIEs), 0.78–5.28 eV of these complexes. Although the IEs of X atoms are high, the AIEs of X@3^6^adz (X = H, B, C, N, and Si) are even lower than the IE of the Cs atom (3.89 eV), which identifies their non-metallic superalkali characteristics. Furthermore, the presence of diffuse excess electron in B@3^6^adz enables it not only to possess a fairly low AIE of 2.16 eV but also significantly high first hyperpolarizability (*β*_0_) of 1.35 × 10^6^ au. This may suggest its potential to be a bifunctional molecule with both strong reducibility and larger NLO response.

Cyraniak et al. adopted the electronic transmutation concept for designing novel series of polynuclear superhalogen anions. They investigated the stability of (BF_3_(BN)_*n*_F_4*n*+1_)^−^ anions for *n* = 1–10, 13, 18–20 having alternate boron and nitrogen central atoms in the form of the (BN)_*n*_ “core”, 4*n*+1 fluorine as well as one BF_3_ ligand using density functional theory (DFT) and outer valence Green function (OVGF) methods with flexible atomic orbital basis sets. The newly proposed anions are reflecting the structures of chain-like C_*n*_H_2*n*+2_ molecules in which the (BN)_*n*_ cores are supposed to resemble the bonding features of the C_2*n*_ chain, which advocates the existence of 4*n*+2 substituents similar to those in typical saturated hydrocarbons. The authors confirmed that the equilibrium configurations of these anions are completely extended chains with each B and N core atom having four peripheral substituents organized in a tetrahedral pattern. Consequently, these anions mimic the universally stable completely extended conformational changes of higher n-alkanes. Moreover, the calculations revealed that the vertical electron detachment energies (VDEs) of the (BF_3_(BN)_*n*_F_4*n*+1_)^−^ anions were observed to be greater than 8 eV and to rise steadily with the increase in *n*. Additionally, the authors predicted that the upper limit of VDE which could be achieved for such polynuclear superhalogen anions is about 10.7 eV.

Guo et al. reported a quantum chemical study on a binary B_5_O_6_^−^ cluster and found a global-minimum planar C_2v_ (^1^A_1_) structure having a B_3_O_3_ hexagonal ring in its center. They noticed that two boronyl (BO) groups and one O^−^ ligand terminates three unsaturated B sites and therefore, formulated this cluster as B_3_O_3_(BO)_2_O^−^ unlike its predecessors, C_s_ B_5_O_5_^−^ and T_d_ B_5_O_4_^−^, containing a tetrahedral B at the center. According to them, the B_5_O_*n*_^−^ series undergoes a significant structural change after oxidation, showing an interesting rivalry among tetrahedral *versus* heterocyclic structures. Their investigations reveal that the B_5_O_6_^−^ cluster has a weak 6*π* aromaticity, making it a boronyl counterpart of the phenolate anion (C_6_H_5_O^−^) or boronyl boroxine. The authors estimated that the B_5_O_6_^−^ cluster has a VDE of 5.26 eV at PBE0 method, which is much higher than the electron affinities of typical halogens, limited to 3.61 eV for Cl, and consequently, declared it as a superhalogen anion.

Zhu et al. studied the structural evolution, bonding, stability, charge transfer, and nonlinear optical characteristics of AuMg_*n*_ (*n* = 2–12) nanoclusters using the DFT method. They unraveled a planar structure of the AuMg_3_ and the symmetrically perfect cage-like structure of the AuMg_9_. They analyzed the charge transfer from the Mg to Au atoms and identified the covalent Mg-Mg bonds in nanoclusters larger than that of AuMg_3_. Their polarizability and hyperpolarizability estimations predict the strong NLO behavior of the AuMg_9_ nanocluster. Based on their theoretical results, the authors also suggested that these nanoclusters are identifiable by spectroscopic experiments.

Tkachenko et al. examined ionization potentials (IPs) for a series of clusters of various cryptand such as [bpy.bpy.bpy]cryptand (bpy = bi-pyridine), [2.2.2]cryptand, and spherical cryptand with Na, K, NH_4_, and H_3_O with by using the DFT as well as *ab initio* methods. They noticed that the encapsulation of Rydberg molecules (NH_4_ and H_3_O) inside an organic cage leads to a decrease in their IPs, reaching the values of ∼1.5 eV and an even lower value of 1.3 eV. They also showed that the Rydberg molecules coated with the “organic skin” can increase their thermodynamic stability. Therefore, they suggested that these findings provide an opportunity to obtain such strong reducing agents in the experiment. This work was contributed by the research group of Prof. Alexander Boldyrev, one of the pioneers in the field of superatoms.

He et al. investigated and studied the substituent effect in M@Al_12_N_11_ and M@Al_11_N_12_ (M = Be, Mg, and Ca) nanocages through the replacement of one Al or N atom of aluminum nitride nanocage (Al_12_N_12_) with an alkaline-earth metal atom by DFT methods. Their calculations reveal that these nanocages are highly stable and have excess electron systems. They, further, suggested that these substituted nanocages exhibit larger first hyperpolarizabilities (*β*_0_) than that of pure Al_12_N_12_ nanocages due to the presence of diffuse excess electrons. According to them, these modified cages possess the transparency to infrared light (IR) (>1,800 nm) as well as ultraviolet light (UV) (250 nm). The authors concluded that their studied highly stable excess electron compounds might be suitable candidates for novel UV and IR NLO systems.

Yang et al. proposed and systematically investigated a new group of hetero-binuclear superhalogen anion matching the MM′X_4_^–^ (M = Li, Na; M′ = Be, Mg, Ca; X = Cl, Br) formula by using second-order Moller-Plesset perturbation theory (MP2) and OVGF methods and 6–311+G (3df) basis set. The calculations revealed that all of the studied anions possess vertical electron detachment energies (VDEs) larger than 5.4 eV and thus confirmed their superhalogen nature. Moreover, the authors also noticed the dependence of the VDE of the MM′X_4_^– ^anions on the M and M′ atomic radius, and electronegativity of the ligand used. In particular, the inclusion of a smaller alkali metal atom M, a bigger alkaline earth metal atom M′, and a greater electronegative ligand atom X might result in larger VDE values for these heteronuclear superhalogen anions. Therefore, one isomer of LiCaCl_4_^–^ was found to possess the largest VDE value (6.799 eV). In addition, the analysis of charge distribution of all studied anions shows that the isomers exhibit considerably greater electrical stability when the excess of electrons charge is shared across all of the ligand atoms as well as with the three bridging ligand atoms.

Thus, the Research Topic not only covers the articles based on the exploration of structures, properties and applications of superatoms but also includes a few articles on atomic clusters as well. We are of the strong opinion that the contents of this Research Topic offer readers an overview of the recent progress of this constantly expanding field.
